# Bioinformatic Analysis of Na^+^, K^+^-ATPase Regulation through Phosphorylation of the Alpha-Subunit N-Terminus

**DOI:** 10.3390/ijms24010067

**Published:** 2022-12-21

**Authors:** Emma-Lucille Blayney, Milna Chennath, Charles G. Cranfield, Ronald J. Clarke

**Affiliations:** 1 School of Chemistry, University of Sydney, Sydney, NSW 2006, Australia; 2 School of Life Sciences, University of Technology Sydney, Ultimo, NSW 2007, Australia; 3 The University of Sydney Nano Institute, Sydney, NSW 2006, Australia

**Keywords:** ion pump, electrostatic switch, mirror tree, protein kinase C, Src kinase, lipid–protein interaction

## Abstract

The Na^+^, K^+^-ATPase is an integral membrane protein which uses the energy of ATP hydrolysis to pump Na^+^ and K^+^ ions across the plasma membrane of all animal cells. It plays crucial roles in numerous physiological processes, such as cell volume regulation, nutrient reabsorption in the kidneys, nerve impulse transmission, and muscle contraction. Recent data suggest that it is regulated via an electrostatic switch mechanism involving the interaction of its lysine-rich N-terminus with the cytoplasmic surface of its surrounding lipid membrane, which can be modulated through the regulatory phosphorylation of the conserved serine and tyrosine residues on the protein’s N-terminal tail. Prior data indicate that the kinases responsible for phosphorylation belong to the protein kinase C (PKC) and Src kinase families. To provide indications of which particular enzyme of these families might be responsible, we analysed them for evidence of coevolution via the mirror tree method, utilising coevolution as a marker for a functional interaction. The results obtained showed that the most likely kinase isoforms to interact with the Na^+^, K^+^-ATPase were the θ and η isoforms of PKC and the Src kinase itself. These theoretical results will guide the direction of future experimental studies.

## 1. Introduction

It has been well established that the trafficking of many peripheral membrane proteins within cells is controlled by an electrostatic switch mechanism that is regulated through phosphorylation by protein kinases [1,2]. The attachment of peripheral membrane proteins to the cytoplasmic face of the plasma membrane occurs through two means. One is via the hydrophobic effect. Many peripheral membrane proteins undergo post-translational modification, whereby a hydrocarbon chain, such as myristoyl or palmitoyl, is added either to the protein’s N- or C-terminus. The hydrocarbon chain inserts itself into the lipid matrix of the membrane and acts as a hydrophobic anchor to the membrane. This interaction alone, however, is insufficient to hold the protein onto the membrane. In addition, the peripheral membrane proteins undergo an electrostatic interaction with the membrane which is mediated by the positively charged lysine or arginine residues on either the N- or C-terminus of the protein and the negatively charged phospholipid headgroups, such as phosphatidylserine, on the cytoplasmic membrane surface. The release of such a peripheral membrane protein from the membrane and its trafficking to another location in the cell is controlled through the regulatory phosphorylation of the protein’s N- or C-terminus by a protein kinase. Phosphorylation introduces between one and two negative charges (depending on the pH) onto the protein chain and, thus, at least partially neutralises the positive charges of the protein’s lysine and arginine residues. Thus, the interaction with the negatively charged membrane surface is weakened, and the protein is released from the membrane; thus, the term “electrostatic switch” was created.

**Figure 1:**
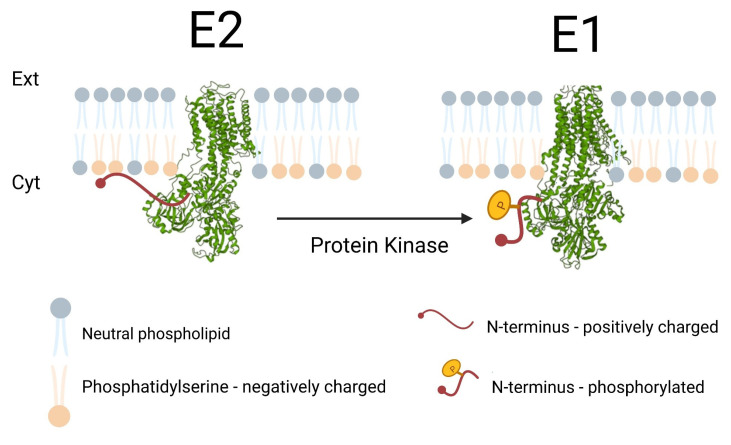
Proposed operation of the electrostatic switch mechanism in the regulation of the Na^+^, K^+^-ATPase. The E2 conformation of the enzyme is stabilised through an electrostatic interaction between positively charged lysine residues on the N-terminus and the negatively charged headgroups of phosphatidylserine on the cytoplasmic surface of the surrounding membrane. Phosphorylation of hydroxyl groups of conserved serine and tyrosine residues of the N-terminus by protein kinases introduces negative charges onto the N-terminus, weakening its electrostatic interaction with the membrane and causing its release from the membrane. This destabilises the E2 conformation of the protein and facilitates its conformational change into the E1 state. The E2 and E1 structures shown in the figure were derived from published crystal structures PDB 3B8E in the case of the E2 state [7] and PDB 3WGU in the case of the E1 state [8].

Although this mechanism of regulation is well substantiated for peripheral membrane proteins, which bind transiently to membrane surfaces, it is not yet widely accepted that it could also play a role in the regulation of integral membrane proteins. Of course, integral membrane proteins do not migrate within a cell but stay embedded in the membrane with which they are associated. Therefore, an electrostatic switch mechanism would not be involved in the trafficking of an integral membrane protein, but it could be involved in the regulation of the protein’s activity. A clear candidate is the Na^+^, K^+^-ATPase which possesses a positively charged lysine-rich N-terminal tail located on the cytoplasmic surface of the protein and which also contains conserved tyrosine and serine residues that are both potential targets for regulatory phosphorylation by protein kinases [3]. The postulated mechanism is shown schematically in Figure 1. Because the Na^+^, K^+^-ATPase ion pumping cycle involves a series of transitions between unphosphorylated and autophosphorylated states, i.e., repeating cycles of E2 → E1 → E1P → E2P → E2, the blockage of the enzyme in any of these conformational states would lead to its inhibition. Under normal physiological conditions, however, simulations of the pump cycle based on the kinetic parameters determined for the individual partial reactions have shown that the E2 → E1 transition is a major rate-determining step of the entire cycle [4]. Therefore, kinase-based regulatory phosphorylation which favours the E1 state over E2 would be expected to significantly enhance the ion pumping activity. Situations where enhanced ion pumping activity would be required include those that occur after an action potential in nerve and muscle cells to re-establish the resting membrane potential or those that occur in all cells upon exposure to hypo-osmotic conditions to avoid cell swelling [5,6]. 

The Na^+^, K^+^-ATPase belongs to the P-type ATPase family of enzymes, a large group of integral membrane proteins which utilize the energy released through the hydrolysis of ATP to pump either small ions or lipids across the membrane in which they are embedded. The Na^+^, K^+^-ATPase belongs to the P2C group of the P2 subfamily of P-type ATPases. It plays crucial roles in animal cell physiology. The Na^+^ electrochemical potential gradient created across the plasma membrane of animal cells by the Na^+^, K^+^-ATPase (or sodium pump) is used to drive nutrient reabsorption in the kidneys, and both the Na^+^ and K^+^ gradients that it generates are essential for nerve and muscle function [9].

The two residues on the N-terminus of the catalytic α_1_-subunit of the Na^+^, K^+^-ATPase which are potential targets for regulatory phosphorylation are tyrosine-5 (Tyr-5) and serine-11 (Ser-11), where the numbering is based on the *Homo sapiens* sequence and excludes the protein’s propeptide sequence (MGKGV in humans). Tyr-5 (or Tyr-10 if the propeptide sequence is included in the numbering) has been postulated to be a target for phosphorylation by the members of the Src kinase family and to play an important role in the regulation of Na^+^, K^+^-ATPases in the kidneys [10]. Ser-11 (or Ser-16 if the propeptide is included) has been found in in vitro experiments on purified kidney Na^+^, K^+^-ATPases [11] and in oocyte homogenates expressing the Na^+^, K^+^-ATPases of the frog *Bufo marinus* [12] to be a target for phosphorylation by protein kinase C (PKC). However, support for the in vivo regulation of the Na^+^, K^+^-ATPase by these kinases is still lacking. The principle on which the research presented here is based is that if two proteins which are still undergoing evolution interact with one another, it is likely that they are coevolving, in part so that the mutations in one protein are compensated for by the mutations in the other protein to optimize their interaction [13,14]. Coevolution analysis has been successfully used elsewhere to identify interacting proteins [15,16]. Therefore, to find evidence for a functional interaction between the Na^+^, K^+^-ATPase and regulatory kinases, we carried out an analysis of the coevolution of the Na^+^, K^+^-ATPase with the isoforms of PKC and the Src kinase family via two methods: mirror tree analysis and phylogenetic distribution analysis [13,17].

PKC possesses ten isoforms that are categorised into three subgroups as determined by their activation and structure: conventional PKCs (α, β1, β2, and γ) which require both Ca^2+^ and diacylglycerol for activation, novel PKCs (δ, ε, η, and θ) which require diacylglycerol for activation but are insensitive to Ca^2+^, and atypical PKCs (ζ and λ(ι)) which are insensitive to both Ca^2+^ and diacylglycerol. Which particular isoform could be responsible for the regulation of the Na^+^, K^+^-ATPase is unknown. Similarly, there are nine members of the Src kinase family which are divided into three subfamilies: the SrcA subfamily consists of Src, Yes, Fyn, and Fgr; the SrcB subfamily consists of Lck, Hck, Blk, and Lyn; and Frk is the only member in the third subfamily. Potentially any of these members of the Src family of the nonreceptor tyrosine kinases could regulate the Na^+^, K^+^-ATPase. Therefore, in the bioinformatic analyses presented here, we investigated the potential coevolution of the Na^+^, K^+^-ATPase with each of the members of the PKC and Src kinase families to discover which members of these families are the ones most likely to interact with the Na^+^, K^+^-ATPase.

## 2. Results

### 2.1. Mirror Tree Analysis of the Na^+^, K^+^-ATPase with PKC Isoforms

**Figure 2:**
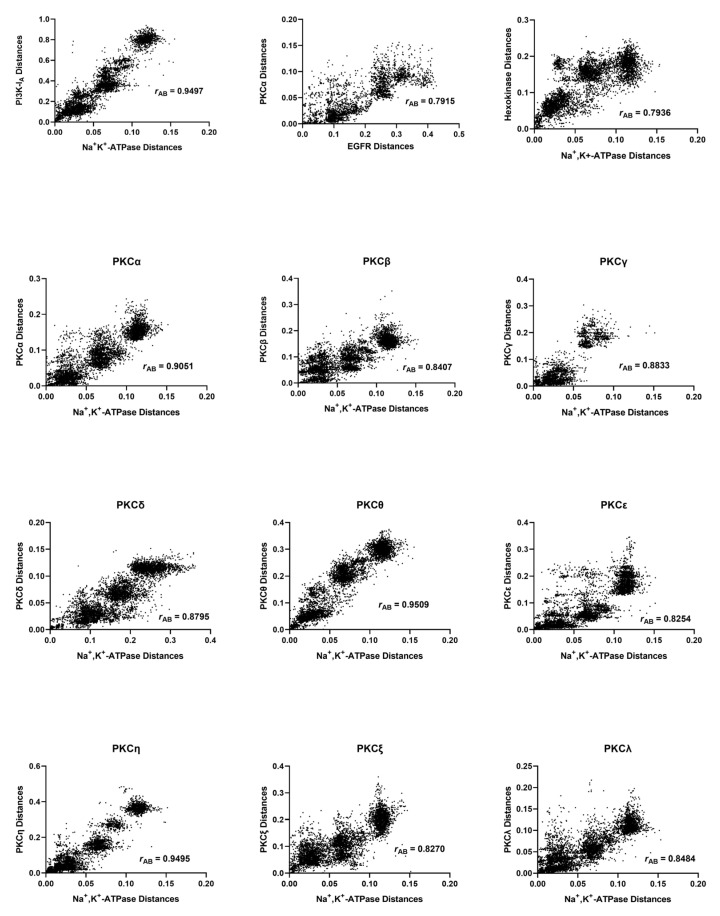
Correlations of evolutionary distances between common organisms expressing various isoforms of PKC and the Na^+^, K^+^-ATPase α_1_-subunit. Each data point represents a combination of a pair of common organisms for each protein as explained in the Methods section. Hence, there are many more data points than common organisms. For comparison, the top row of the figure shows correlations of the positive control, PI3K-I_A_, with the Na^+^, K^+^-ATPase followed by two negative controls, i.e., PKCα, with EGFR followed by hexokinase with the Na^+^, K^+^-ATPase.

Mirror tree analyses of the Na^+^, K^+^-ATPase α_1_ isoform with each of the isoforms of PKC were conducted. Correlations of the pairwise distances are shown in Figure 2. The corresponding Pearson correlation coefficients, *r*_AB_; the number of common organisms, *N*; and the number of pairings of the common organisms (i.e., the number of data points in the correlation plots), *n*, are given in Table 1.

The highest correlation coefficients between the Na^+^, K^+^-ATPase and the PKC isoforms were 0.951 and 0.950 for the θ and η isoforms, respectively. The probability, *p*, of the null hypothesis that the correlation of the Na^+^, K^+^-ATPase with the η isoform is no greater than that with the θ isoform was 0.424. Therefore, based on this data alone, there was no strong evidence favouring the coevolution of the Na^+^, K^+^-ATPase with the θ isoform over the η isoform. In contrast, the correlation with the θ isoform was found to be significantly higher than that of all the other isoforms (α, β, γ, δ, ε, ζ, and λ), with a *p* value of <0.0001. It should be noted, however, that PKCβ underwent alternative splicing to give two unique isoforms, PKCβ1 and PKCβ2. However, because the UniProt database, which we used to obtain the protein sequences, does not distinguish between these two isoforms, the analysis presented here treated PKCβ as a single isoform. The results obtained for PKCβ should, therefore, be treated with some caution.

### 2.2. Mirror Tree Analysis of the Na^+^, K^+^-ATPase with the Enzymes of the Src Kinase Family

**Figure 3:**
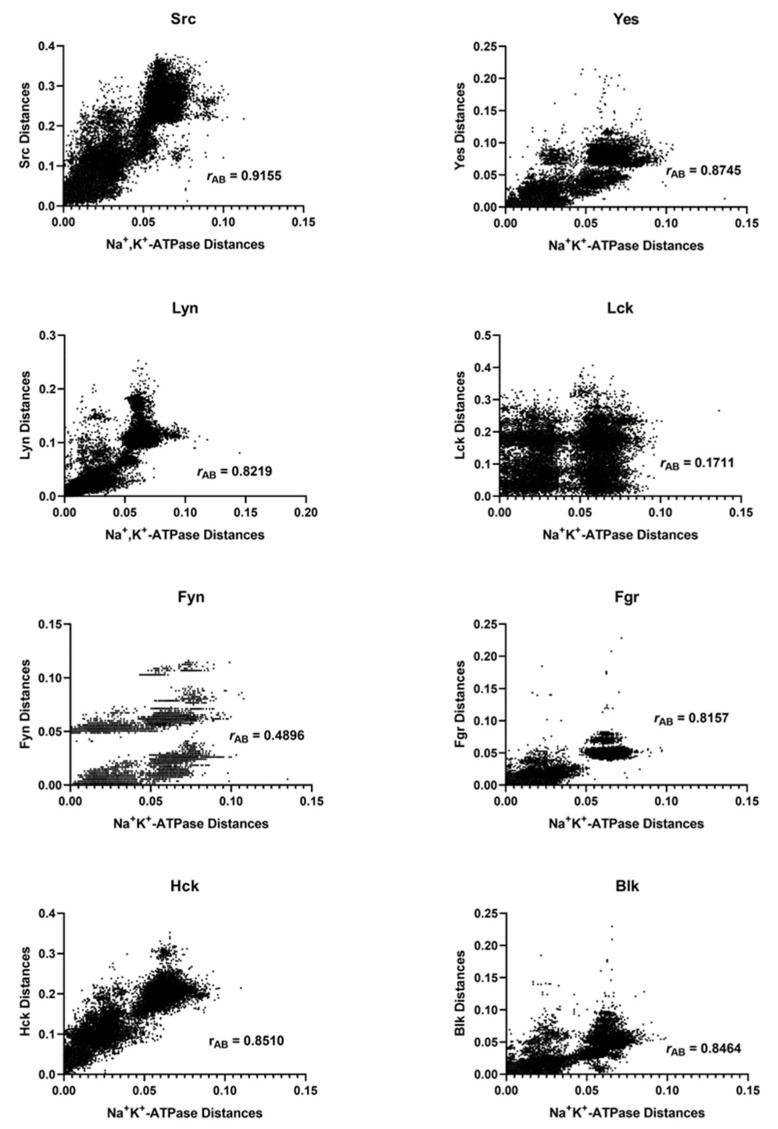
Correlations of evolutionary distances between common organisms that express different members of the Src kinase family of enzymes and the Na^+^, K^+^-ATPase α_1_-subunit.

Mirror tree analyses of the Na^+^, K^+^-ATPase α_1_ isoform with each member of the Src kinase family were also conducted. The correlations of the pairwise distances are shown in Figure 3. The corresponding Pearson correlation coefficients, *r*_AB_; the number of common organisms, *N*; and the number of pairings of the common organisms (i.e., the number of data points in the correlation plots), *n*, are given in Table 2.

The highest correlation coefficient between the Na^+^, K^+^-ATPase and the members of the Src kinase family was 0.9155 for the Src kinase. The correlation with the Src kinase was found to be significantly higher than that of all the other members of the family (Yes, Lyn, Lck, Fyn, Fgr, Hck, and Blk), with a *p* value of< 0.0001.

### 2.3. Comparisons of the Phylogenetic Distributions of the Na^+^, K^+^-ATPase with PKC Isoforms

**Figure 4:**
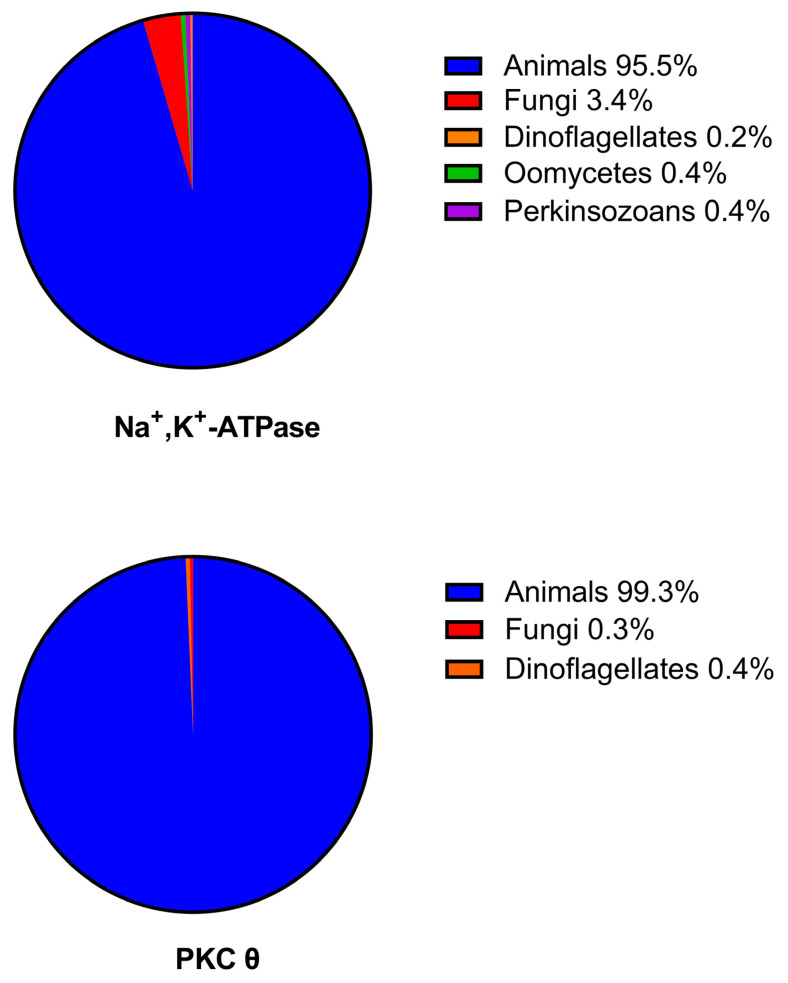
Phylogenetic distributions of the Na^+^, K^+^-ATPase α_1_-subunit and PKCθ.

The phylogenetic distributions of the Na^+^, K^+^-ATPase α_1_ subunit and PKCθ are shown in Figure 4. It can be seen that both the Na^+^, K^+^-ATPase and PKCθ were predominantly distributed amongst animals. The distribution of PKCθ was shown because this was the isoform which showed the strongest evidence of coevolution with the Na^+^, K^+^-ATPase via the mirror tree analysis. However, all the other PKC isoforms showed similar distributions, with over 99% of the distributions occurring in animals. The similarity in the phylogenetic distributions of the Na^+^, K^+^-ATPase and PKCθ were consistent with coevolution, but the similar distributions of all the PKC isoforms did not allow any conclusions to be drawn regarding the particular isoform that was the most likely regulatory reaction partner of the Na^+^, K^+^-ATPase. It would be feasible to inspect the distributions of the Na^+^, K^+^-ATPase and the PKC isoforms across different animal classes. However, because the Na^+^, K^+^-ATPase is known to be present in all animal species, this would provide no evolutionary information. It would merely reflect the relative numbers of each animal class in the database. Therefore, the mirror tree analysis in this case provided more useful results.

The situation of the members of the Src kinase family would seem to be similar. It is already known that its members are expressed in all eukaryotic organisms [18]. This is also consistent with the distribution of the Na^+^, K^+^-ATPase, thus justifying a hypothesis of possible coevolution and interaction between the proteins, but it did not allow information to be determined regarding which member of the Src kinase family was most likely to interact with the Na^+^, K^+^-ATPase.

## 3. Discussion

The purpose of this study was to identify the kinases which are most likely to regulate the activity of the Na^+^, K^+^-ATPase via an electrostatic switch mechanism in which the interaction of the lysine-rich N-terminus with the negatively charged cytoplasmic surface of the surrounding membrane is modulated through the phosphorylation of the hydroxyl groups of the N-terminal tail. Two potential conserved amino acid residues and two potential families of kinases were identified. Ser-11 (or 16 if one counts the propeptide sequence) is a potential target of the serine/threonine-specific kinase protein kinase C, whereas Tyr-5 (or 10 if one counts the propeptide sequence) is a potential target for phosphorylation by the tyrosine kinases of the Src family. The question that we were attempting to answer was which particular isoform or member of these kinase families is the most likely interaction partner of the Na^+^, K^+^-ATPase. The approach we took was to analyse for coevolution. The logic behind this was that if two proteins are interacting, then they should also be coevolving. A simple analogy is that of a lock and a key. Both fit together perfectly, but if the owner of a house changes the locks, they will not be able to access their house anymore unless they also get new keys. Thus, when one protein undergoes a mutation, an interacting protein must undergo a compensating mutation to maintain an optimal interaction.

Of all ten PKC isoforms, the θ isoform showed the strongest evidence of coevolution with the Na^+^, K^+^-ATPase via the mirror tree analysis. PKCθ is an isoform found predominantly in the haematopoietic cells [19], i.e., the stem cells, which can develop into all types of blood cells. PKCη displayed a slightly lower correlation than PKCθ, but the difference was not statistically significant. PKCη is an isoform expressed predominantly in epithelial cells [20].

Amongst the Src kinase family of enzymes, the Src kinase itself was found to have the highest correlation with the Na^+^, K^+^-ATPase. The Src kinase is known to interact with membranes [21], in particular the plasma membrane, where the Na^+^, K^+^-ATPase is also located. At its N-terminus, the Src kinase undergoes a post-translational myristoylation. The fatty acid tail of the myristoyl group inserts itself into membranes and acts as a hydrophobic anchor to the membrane. This would be expected to enhance its phosphorylation of membrane proteins because the diffusion of the Src kinase to its membrane protein target would then be a two-dimensional surface diffusion rather than diffusion in a three-dimensional volume.

In conclusion, it is important to bear in mind that the correlations observed here in the evolution of the Na^+^, K^+^-ATPase α_1_ subunit and the θ and η isoforms of PKC as well as with the Src kinase cannot be seen as definitive proof that the Na^+^, K^+^-ATPase is regulated by these enzymes. However, considering the number of different enzymes in the PKC and Src kinase families, it is useful to have an indication of the particular isoforms which seem to be the most likely candidates for Na^+^, K^+^-ATPase regulatory kinases for the purposes of guiding future experimental research. Nevertheless, the observation of the correlations supporting the coevolution of the Na^+^, K^+^-ATPase and PKCθ, PKCη, and Src is, as far as we were aware, the first indication that regulation via these kinases is occurring in vivo. 

## 4. Methods

### 4.1. Mirror Tree Analysis

One way to analyse the degree of coevolution and, hence, to identify interaction partners, i.e., in this case, enzymes which could regulate the Na^+^, K^+^-ATPase, is to use the mirror tree method, which analyses the degree of similarity between two proteins’ phylogenetic trees [22,23,24].

Amino acid sequences of pairs of proteins of interest (e.g., the Na^+^, K^+^-ATPase and different isoforms of protein kinase C (PKC) which phosphorylate serine (S) or threonine (T) residues) were obtained from the UniProt protein database using a stringent BLAST search (e-value ≤ 0.0001) and by inputting the Homo sapiens sequence of the protein of interest and filtering for vertebrates. The top 500 hits from the search for each protein were collected. To determine whether PKC could interact with the N-terminus of the Na^+^, K^+^-ATPase, each of the isoforms of PKC (α, β, γ, δ, ε, η, θ, ζ, and λ (alternatively known as ι)) were analysed against the catalytic α-subunit (containing the N-terminus) of the Na^+^, K^+^-ATPase. Two negative controls were used: (1) Hexokinase (a carbohydrate kinase, not a protein kinase) with Na^+^, K^+^-ATPase and (2) PKCα with epidermal growth factor receptor (EGFR), a protein that is not phosphorylated by serine/threonine kinases such as PKC [25]. For the positive control, class I_A_ phosphoinositide-3 kinase (PI3K-I_A_) was used as it has previously been shown to interact with the proline-rich region of the N-terminus of Na^+^, K^+^-ATPase [26].

For the two proteins of interest, the collections of amino acid sequences were filtered to find common species. This typically returned approximately 100 common species for each pair of proteins to be analysed. The filtered amino acid sequences were aligned using the MUSCLE program [27] within the MEGA-X suite of evolutionary genetics programs [28], and the evolutionary distances, or pairwise distances, were calculated. The pairwise distance represents the number of amino acid substitutions per site between two homologous proteins, i.e., the number of substitutions divided by the total number of amino acids in the sequence. In terms of a phylogenetic tree, the pairwise distance is defined as the sum of the lengths of each branch of the tree connecting two species. The complete set of pairwise distances for each protein was exported as a distance matrix. A correlation coefficient, *r_AB_*, between the pairwise distances of each protein was calculated via the method of Pearson as described by the following equation:

(1) $$r_{AB}=\frac{\sum_{i=1}^{n-1}\sum_{j=i+1}^{n}\left(dA_{ij}-\bar{dA_{ij}}\right)\left(dB_{ij}-\bar{dB_{ij}}\right)}{\sqrt{\sum_{i=1}^{n-1}\sum_{j=i+1}^{n}{\left(dA_{ij}-\bar{dA_{ij}}\right)}^{2}}\sqrt{\sum_{i=1}^{n-1}\sum_{j=i+1}^{n}{\left(dB_{ij}-\bar{dB_{ij}}\right)}^{2}}}$$

where *dA_ij_* is the pairwise distance between species *i* and *j* for protein A, *dB_ij_* is the corresponding pairwise distance for protein B, the terms with bars represent the average values of *dA_ij_* and *dB_ij_* across all pairs of species common to both proteins, and *n* is the number of pairings of the common organisms (or the number of elements in a matrix of the common organisms). The value *n* is related to the number of common organisms, *N*, by *n* = (*N*^2^ − *N*)/2. The closer the value of *r_AB_* is to 1, the higher the likelihood that the two proteins coevolved. More precisely, if there is a high correlation, it means that there is a tight relationship between changes in evolutionary rates of the two families of proteins along different branches of their phylogenetic trees. For example, if one protein experiences a rate increase along one branch, then the other protein also experiences a rate increase along the same branch. Shared patterns of rate variation suggest that the proteins have evolved in a correlated manner, perhaps due to similar adaptive pressures.

To compare correlation coefficients obtained for different pairs of proteins, we first converted the *r_AB_* values using Fisher’s r-to-z transformation to obtain a normal distribution. The Fisher *z* coefficient is related to *r_AB_* by:

(2) $$z=\frac{1}{2}\ln\left(\frac{1+r_{AB}}{1-r_{AB}}\right)$$

The two-tailed probability of the null hypothesis, *p*, that two correlation coefficients were not significantly different was then calculated from the corresponding z values and the associated number of pairs of organisms, *N*, using the online Vassarstats website for statistical computation (http://vassarstats.net/) via the tool “Significance of the Difference between Two Correlation Coefficients”. Analysis of individual correlation coefficients was also carried out using the Vassarstats website via the tool “Significance of a Correlation Coefficient”. In this case, *p* represents the probability of the nondirectional null hypothesis that there was no significant correlation between the evolutionary distances of the two proteins being compared.

### 4.2. Phylogenetic Distribution Analysis

A further potential indicator of coevolution of two proteins is their co-occurrence across large numbers of organisms [17]. For example, if one lineage of organisms lost one of the proteins but not the other, this would be inconsistent with coevolution. Therefore, to further analyse whether correlations of evolutionary distances between two protein families identified via the mirror tree analysis could be attributed to a functional coevolution, we carried out an analysis of the phylogenetic distributions of each protein family. Phylogenetic distribution can simply be expressed as the percentage of occurrence of a protein across different phylogenetic classes of organisms.

Phylogenetic distributions profiles were determined by searching for each protein of interest via the National Center for Biotechnology Information (NCBI) website www.ncbi.nlm.nih.gov/protein/ and by utilising the website’s taxonomic group output. For the Na^+^, K^+^-ATPase α_1_ subunit, the search was conducted via the protein’s gene name, ATP1A1, and by restricting the search to amino acid sequence lengths of 1010–2000 to avoid the inclusion of partial sequences. The search for PKC isoforms was also conducted using their corresponding gene names, i.e., PRKCA (α), PRKCB1 (β1), PRKCB2 (β2), PRKCG (γ), PRKCD (δ), PRKCE (ε), PRKCH (η), PRKCQ (θ), PRKCZ (ζ), and PRKCI (λ or ι). The Src kinase is already known to be ubiquitously expressed in vertebrate cells [18].

## Figures and Tables

**Table 1:** Pearson correlation coefficients, *r*_AB_, of mirror tree analyses of coevolution of the Na^+^, K^+^-ATPase α_1_ subunit and isoforms of PKC. *N* is the number of common organisms, and *n* is the number of pairwise combinations of the common organisms.

PKC Isoform	*r*_AB_ *	*N*	*N*
α	0.9051	113	6328
β	0.8407	112	6216
γ	0.8833	81	3240
δ	0.8795	122	7381
ε	0.8254	116	6670
η	0.9495	106	5565
θ	0.9509	118	6903
ζ	0.8270	107	5671
λ	0.8484	113	6328

* In each case, the probability, *p*, of the null hypothesis that there is no significant correlation was <0.0001.

**Table 2:** Pearson correlation coefficients, *r*_AB_, of mirror tree analyses of coevolution of the Na^+^, K^+^-ATPase α_1_ subunit and members of the Src kinase family. *N* is the number of common organisms, and *n* is the number of pairwise combinations of the common organisms.

Src Family Member	*r*_AB_ *	*N*	*N*
Src	0.9155	295	43,365
Yes	0.8745	271	36,585
Lyn	0.8219	303	45,753
Lck	0.1711	248	30,628
Fyn	0.4896	232	26,796
Fgr	0.8157	231	26,565
Hck	0.8510	262	34,191
Blk	0.8464	258	33,153

* In each case, the probability, *p*, of the null hypothesis that there is no significant correlation was <0.0001.

## Data Availability

The authors declare that all required data have been presented in the manuscript. The datasets did not contain any software that needed to be archived.
